# FGF9 from cancer-associated fibroblasts is a possible mediator of invasion and anti-apoptosis of gastric cancer cells

**DOI:** 10.1186/s12885-015-1353-3

**Published:** 2015-04-30

**Authors:** Chao Sun, Hirokazu Fukui, Ken Hara, Xinxing Zhang, Yoshitaka Kitayama, Hirotsugu Eda, Toshihiko Tomita, Tadayuki Oshima, Shojiro Kikuchi, Jiro Watari, Mitsuru Sasako, Hiroto Miwa

**Affiliations:** 1 Division of Gastroenterology, Department of Internal Medicine, Hyogo College of Medicine, l-1, Mukogawa, Nishinomiya, 663-8501 Japan; 2 Department of Digestive Diseases, Tianjin Medical University General Hospital, Tianjin, China; 3 Department of Geriatric Digestive Internal Medicine, Sichuan Academy of Medical Science & Sichuan People’s Hospital, Chengdu, China; 4 Department of Surgery, Hyogo College of Medicine, Nishinomiya, Japan

**Keywords:** FGF, Cancer-associated fibroblast, Invasion, Anti-apoptosis, ERK, Akt, Gastric cancer

## Abstract

**Background:**

Cancer-associated fibroblasts (CAFs), which reside around tumor cells, are suggested to play a pivotal role in tumor progression. Here we performed microarray analyses to compare gene expression profiles between CAFs and non-cancerous gastric fibroblasts (NGFs) from a patient with gastric cancer and found that *fibroblast growth factor 9* (*FGF9*) was a novel growth factor overexpressed in CAFs. We then examined the biological effects of FGF9 during progression of gastric cancer.

**Methods:**

Expression of FGF9 in CAFs and NGFs, and their secreted products, were examined by Western blotting. The effects of FGF9 on AGS and MKN28 gastric cancer cells in terms of proliferation, invasion and anti-apoptosis were assessed by WST-1 assay, invasion chamber assay and FACS, respectively. Furthermore, the intracellular signaling by which FGF9 exerts its biological roles was examined *in vitro*.

**Results:**

FGF9 was strongly expressed in CAFs in comparison with NGFs, being compatible with microarray data indicating that FGF9 was a novel growth factor overexpressed in CAFs. Treatment with FGF9 promoted invasion and anti-apoptosis through activation of the ERK and Akt signaling pathways in AGS and MKN28 cells, whereas these effects were attenuated by treatment with anti-FGF9 neutralizing antibody. In addition, FGF9 treatment significantly enhanced the expression of matrix metalloproteinase 7 (MMP7) in both cell lines.

**Conclusions:**

FGF9 is a possible mediator secreted by CAFs that promotes the anti-apoptosis and invasive capability of gastric cancer cells.

**Electronic supplementary material:**

The online version of this article (doi:10.1186/s12885-015-1353-3) contains supplementary material, which is available to authorized users.

## Background

The formation of cancerous lesions is intimately associated with their unique microenvironment. Progression is closely correlated with the capability of cancer cells to recruit and activate the surrounding stromal cells, and subsequently exploit them [1]. Cancer-surrounding stromal cells, such as fibroblasts, endothelial cells, and immune cells, can orchestrate tumorigenesis and metastasis through cell-to-cell interaction and/or production of soluble growth factors/cytokines/chemokines [1-3]. In this context, fibroblasts in cancerous lesions are known as cancer-associated fibroblasts (CAFs) and have received much attention with regard to their role in tumor progression [4,5]. Although CAFs are known to be largely different from normal fibroblasts in non-neoplastic tissues in terms of their gene profile, the mechanism by which CAFs promote tumor progression is unclear. Therefore, we compared the gene expression profiles of CAFs, focusing especially on growth factors/cytokines/chemokines, with those of non-cancerous gastric fibroblasts (NGFs) using microarray assay, and subsequently isolated *fibroblast growth factor 9* (*FGF9*) as a novel gene that was overexpressed in CAFs in gastric cancer.

FGF9, a secretory protein of the FGF family, is reportedly expressed in stromal cells including fibroblasts [6-8]. In general, FGF signaling occurs via FGF receptors (FGFRs) to regulate a variety of cell biological behavior, including proliferation, differentiation, survival and motility [9], and expression of FGF9, FGFR2c, FGFR3b and FGFR3c has been detected in gastric and colon cancers [10]. Thus, like other FGF family proteins, FGF9 may play a pivotal role in the interaction between cancer cells and their surrounding stromal cells, and it is noteworthy that FGF9 is strongly expressed in CAFs in gastric cancer. In the present study, we screened for differences in gene expression between CAFs and NGFs from a patient with gastric cancer. We examined the effect of FGF9 on proliferation, invasion and anti-apoptosis of gastric cancer cells, and moreover clarified the intracellular signaling by which FGF9 exerts its biological effects on gastric cancer cells.

## Methods

### Reagents and cell culture

Human recombinant FGF9 and anti-human FGF9 neutralizing antibody were purchased from R&D Systems (Minneapolis, MN, USA). Anti-extracellular signal-regulated protein kinase (ERK), anti-phospho-specific ERK (p-ERK), anti-Akt, anti-phospho-specific Akt (p-Akt; Ser473), and anti-β-actin antibodies were purchased from Cell Signaling Technology (Beverly, MA, USA).

The gastric cancer cell lines AGS was cultured in Ham’s F-12 medium (Sigma, Aurora, Ohio, USA) with 10% fetal bovine serum (FBS; Biowest, Nuaillé, France) in a humidified incubator at 37°C with an atmosphere of 5% CO_2_. Similarly, MKN28 was cultured in RPMI 1640 medium with 10% fetal bovine serum, and other five gastric cancer cell lines MKN1, MKN 45, MKN74, GCIY, and KATOIII were maintained as previously described [11].

### Isolation and culture of human gastric fibroblasts

Human gastric cancer (poorly-differentiated adenocarcinoma) specimens were obtained from a patient who underwent gastrectomy at Hyogo College of Medicine Hospital in 2012. Cancer-associated fibroblasts (CAFs) were prepared from the cancerous portion in the stomach. Non-cancerous gastric fibroblasts (NGFs) were prepared from non-cancerous portion with atrophic gastritis at least 50 mm far from tumor in the stomach. The tissue specimens were trimmed of fat and necrotic tissue, minced with scalpels and washed in PBS containing antibiotic-antimycotic reagent (Anti-Anti®, GIBCO). The tissue pieces were transferred to a 12-well microplate (IWAKI, Tokyo, Japan) at one fragment/well. The cells were cultured in DMEM medium (GIBCO, Grand Island, NY, USA) with 10% heat-inactivated FBS at 37°C in an atmosphere of 5% CO2. The fibroblasts that initially grew in a monolayer were collected, transferred to another dish and used for experiments within the 10th passage. These studies were done with the approval of the Review Board of Hyogo College of Medicine, and informed consent was obtained from the patient.

### Microarray analysis

Using Trizol reagent (Invitrogen, Carlsbad, CA, USA), total RNA was extracted from three sets of CAFs and NGFs cultured. cDNA labeling, hybridizations, scanning and data analysis were performed by Hokkaido System Science Co., Ltd (Sapporo, Japan). Briefly, cyanine-3 (Cy3)-labeled cRNA was prepared from total RNA (0.05 μg) using a Low Input Quick Amp Labeling Kit (Agilent) in accordance with the manufacturer’s instructions, followed by RNAeasy column purification (QIAGEN, Valencia, CA). Dye incorporation and cRNA yield were checked with a NanoDrop ND-1000 Spectrophotometer. Cy3-labeled cRNA (0.60 μg) was fragmented at 60°C for 30 min in a reaction volume of 25 μl containing 1x Agilent fragmentation buffer and 2x Agilent blocking agent in accordance with the manufacturer’s instructions. On completion of the fragmentation reaction, 25 μl of 2x Agilent hybridization buffer was added to the fragmentation mixture and hybridized to Agilent SurePrint G3 Human Gene Expression Microarray (8x60K ver.2.0) for 17 h at 65°C in a rotating Agilent hybridization oven. After hybridization, the microarrays were washed for 1 min at room temperature with GE Wash Buffer 1 (Agilent) and for 1 min at 37°C with GE Wash buffer 2 (Agilent), then dried immediately by brief centrifugation. Slides were scanned immediately after washing on an Agilent DNA Microarray Scanner (G2565CA) using one color scan setting for 8x60K array slides (Scan Area 61×21.6 mm, Scan resolution 3μm, Dye channel for Green PMT set to 100%). The scanned images were analyzed and normalized with Feature Extraction Software 10.7.3.1 (Agilent).

### RNA extraction and reverse transcription-polymerase chain reaction (RT-PCR)

Total RNA was extracted from gastric cancer cell lines using Trizol reagent (Invitrogen). Four microgram of total RNA was reverse-transcribed by using oligo dT (Applied Biosystems, Branchburg, NJ, USA) and 200 U of Superscript™ II reverse transcriptase (Invitrogen) in a total volume of 20μl. For the following PCR, pairs of oligonucleotide primers for *human FGFRs* were prepared as previously described [12]. Human *FGFR2c*: 5′-TGGTCGGAGGAGACGTAGAG-3′ (Forward) and 5′-AAAGTTACATTCCGAATATAGAGAACC-3′ (Reverse); human *FGFR3b*: 5′-GGAGTTCCACTGCAAGGTGT-3′ (Forward) and 5′ -GTGAACGCTCAGCCAAAAG-3′ (Reverse); human *FGFR3c*: 5′-GGAGTTCCACTGCAAGGTGT-3′ (Forward) and 5′-AAGCGGGAGATCTTGTGC-3′ (Reverse); human *GAPDH*: 5′-GGCTGCTTTTAACTCTGGTA-3′ (Forward) and 5′-ATGCCAGTGAGCTTCCCGT-3′ (Reverse). One microliter of RT product (cDNA) was amplified by PCR in a 50-μl reaction volume containing 20 pmol of the above sets of primers, 1.25 U of Ampli-Taq DNA polymerase (Applied Biosystems, Foster City, Calif., USA), and the final PCR buffer: 20 mM Tris–HCl (pH 8.4), 50 mM KCl, 2.5 mM MgCl2, 10 mM dithiothreitol, and 1 mM dNTP. The PCR amplification was performed as follows: for *FGFRs*, at 95°C for 5 min once; 40 cycles at 95°C for 30 s, at 57°C for 30 s, and at 72°C for 1 min; then at 72°C for 7 min; for *GAPDH*, at 95°C for 7 min once; 40 cycles at 95°C for 30 s, at 55°C for 1 min, and at 72°C for 30 sec; then at 72°C for 7 min.

### Real-time RT-PCR

Real-time RT-PCR was performed using 7900H Fast Real-Time PCR System (Applied Biosystem) as previously described [13]. The following sets of primers for human *matrix metalloproteinase 2* (*MMP2*), *MMP3*, *MMP7*, *MMP9*, and *GAPDH* were prepared (Additional file 1: Table S1). Real-time RT-PCR assays were carried out with 200 ng RNA equivalent cDNA, SYBR Green Master Mix (Applied Biosystems), and 500 nmol/l gene specific primers. The PCR cycling conditions were 50°C for 15 s, and 60°C for 60 s. The intensity of the fluorescent dye was determined, and each of mRNA expression levels was normalized to *GAPDH* mRNA expression levels.

### Cell proliferation assay

AGS (4 × 10^3^) and MKN28 cells (1 × 10^4^) were seeded in complete medium in 96-well microplates. The medium was then replaced with one containing recombinant FGF9 (0—10 ng/ml). WST-1 solution was added after 72 h incubation, and the plates were incubated at 37°C for 1 h. The plates were analyzed using an ELISA plate reader at 450 nm with the reference wavelength set at 600 nm.

### Cell invasion assay

Cell invasion assay was performed using BioCoat Matrigel invasion chambers (BD Biosciences, Bedford, MA, USA) according to the manufacturer’s protocol. Briefly, AGS cells (1 × 10^5^) or MKN28 cells (3 × 10^5^) were seeded in the insert of the Matrigel-coated invasion chamber (24 wells, 8-μm pore size) filled with serum-free medium containing different concentrations of FGF9 (0–10 ng/ml). Then, the cells were incubated with medium containing 10% FBS in the lower chamber at 37°C in 5% CO2. To inhibit the effects of FGF9, anti-FGF9 antibody (1 μg/ml) was also added to the upper chamber. After incubation for 27 h, non-invading cells were removed using a cotton swab and the cells that had invaded into the lower surface of the membrane were fixed with ethanol. The invading cells were then stained with hematoxylin and counted using a microscope in five different visual fields (magnification, x200).

### Apoptosis assay

AGS (2 × 10^5^) and MKN28 (2.5 × 10^5^) cells were seeded in six-well plates in routine medium for 24 h. The cells were then deprived of serum and treated with or without recombinant FGF9 (1–10 ng/ml) for 48h. To inhibit the effects of FGF9, anti-FGF9 antibody (1 μg/ml) was also added to the culture medium. After treatment, both floating and attached cells were harvested, washed with PBS and stained with AnnexinV-FITC and propidium iodide (PI) using a MEBCYTO Apoptosis Kit (MBL, Nagoya, Japan). Stained cells were analyzed on a FACScalibur flow cytometer (Becton Dickinson, Franklin Lakes, NJ, USA), and the data obtained were analyzed using CELLQUEST software (Becton Dickinson, Mountain View, CA, USA).

### Western blot analysis

Western blot analyses were performed as described previously [14]. Briefly, after treatment with or without reagent, cells were lysed in protein extraction buffer, and protein extract (30 μg) was fractioned by sodium dodecyl sulfate polyacrylamide gel electrophoresis and transferred to a nitrocellulose blotting membrane. The membrane was incubated with a primary antibody and then with a peroxidase-conjugated secondary antibody. Proteins were detected using an enhanced chemiluminescence system (Amersham Biosciences, Buckinghamshire, UK).

### Immunohistochemistry

A total of 20 gastric cancers tissues were obtained from specimens resected surgically at Hyogo College of Medicine. The tissue specimen were fixed in 10% formalin solution and embedded in paraffin. This study was approved by the Review Board of Hyogo College of Medicine, and informed consent was obtained from all patients. The characteristics of gastric cancer patients were showed in Additional file 2: Table S2.

Immunohistochemical staining for FGF9 was performed with an LSAB+ kit using anti-FGF9 antibody (1:40; R&D Systems, Minneapolis, MN, USA) as described previously [15]. Finally, the sections were incubated in 3,3′-diaminobenzide tetrahydrochloride with 0.05% H2O2 for 3 min, and then counterstained with Mayer’s haematoxylin. To evaluate the immunoreactivity of FGF9, at least five different visual fields were observed at the invasive front of gastric cancer lesions. A specimen was considered positive when FGF9-positive fibroblastic nests were observed in the visual fields examined.

### Statistics analysis

All values were expressed as the mean ± SD. The data were analyzed using unpaired two-tailed *t*-test. *P* values of less than 0.05 were considered to indicate statistical significance.

## Results

### Microarray analyses of CAFs in gastric cancer tissues

**Figure 1:**
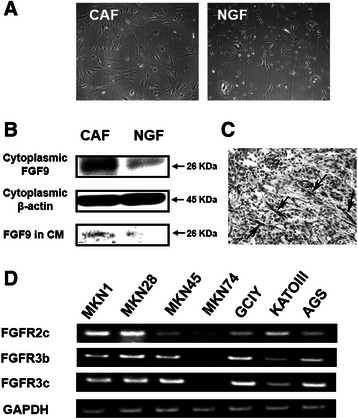
Expression of FGF9 and its receptors in CAFs and gastric cancer cells. **(A)** Morphology of gastric CAFs and NGFs. **(B)** Production of FGF9 in gastric CAFs, NGFs and their conditioned medium (CM). **(C)** Expression of FGF9 in CAFs of the gastric cancer lesion. Arrows indicating CAFs. **(D)** Expression of *FGF receptors* responsible for FGF9 in gastric cancer cell lines.

We isolated CAFs and NGFs (Figure 1A) and compared the gene expression profile of CAFs with that of NGFs using microarray assay. Ten representative genes that were upregulated in CAFs are listed in Table 1. Among these genes, we targeted FGF9 as the most highly expressed gene to examine the role of this CAF-produced growth factor on gastric cancer cells, and in fact before starting *in vitro* studies we confirmed that CAF cells produced much larger amount of FGF9 protein than NGF cells (Figure 1B). Moreover, we confirmed that FGF9 is strongly expressed in the fibroblasts in the stroma of the gastric cancer lesion from which CAF was isolated (Figure 1C).

**Table 1:** **Representative genes differentially expressed in CAFs from NGFs**

Accession No.	Symbol	Gene name	Fold change
**Up-regulated**			
NM_014333	CADM1	Cell adhesion molecule 1, transcript variant 1	273.6
CB178477	XLOC_l2_007424	gb|is39c09.y1 HR85 islet Homo sapiens cDNA clone IMAGE:6554705 5′, mRNA sequence	254.8
NM_001113207	TSTD1	Thiosulfate sulfurtransferase (rhodanese)-like domain containing 1, transcript variant 1	237.5
NM_000867	HTR2B	5-hydroxytryptamine (serotonin) receptor 2B	171.5
NM_001008539	SLC7A2	Solute carrier family 7 (cationic amino acid transporter, y + system), member 2, transcript variant 2	142.5
NM_002010	FGF9	Fibroblast growth factor 9 (glia-activating factor)	141.1
NM_005559	LAMA1	Laminin, alpha 1	119.0
NM_001040058	SPP1	Secreted phosphoprotein 1, transcript variant 1	116.1
A_24_P247454	A_24_P247454	Unknown	112.6
NM_014398	LAMP3	Lysosomal-associated membrane protein 3	111.3
**Down-regulated**			
NM_001141919	XG	Xg blood group (XG), transcript variant 2,	0.0035
NM_175569	XG	Xg blood group (XG), transcript variant 1	0.0044
NM_000609	CXCL12	Chemokine (C-X-C motif) ligand 12 (CXCL12), transcript variant 2	0.0071
NM_014817	TRIL	TLR4 interactor with leucine-rich repeats	0.0099
NM_002839	PTPRD	Protein tyrosine phosphatase, receptor type D, transcript variant 1	0.0138
NR_021485	EGFEM1P	EGF-like and EMI domain containing 1, pseudogene, non-coding RNA	0.0141
NM_198285	WDR86	WD repeat domain 86	0.0145
NM_001164000	MECOM	MDS1 and EVI1 complex locus (MECOM), transcript variant 6	0.0166
NM_004335	BST2	Bone marrow stromal cell antigen 2	0.0168
ENST00000484765	XLOC_002912	Hypothetical LOC100507661 (LOC100507661), miscRNA	0.0172

Fold change values were evaluated as a ratio of normalized CAFs/normalized NGFs.

### Expression of *FGFR2c* and *FGFR3b/c* in gastric cancer cell lines

FGF9 has been reported to show high affinity for the FGFR2c isoform and FGFR3b/c isoforms [12]. Therefore, we examined the expression of these *FGFRs* in various gastric cancer lines using RT-PCR. As shown in Figure 1D, expression of *FGFR2c* was detected in all seven gastric cancer cell lines, whereas expression of *FGFR3b/c* was detected in six of the seven, with the exception of MKN74. These findings suggested that gastric cancer cells have the capacity to respond to FGF9 stimulation.

### FGF9 activates the ERK and AKT signaling pathways in gastric cancer cells

**Figure 2:**
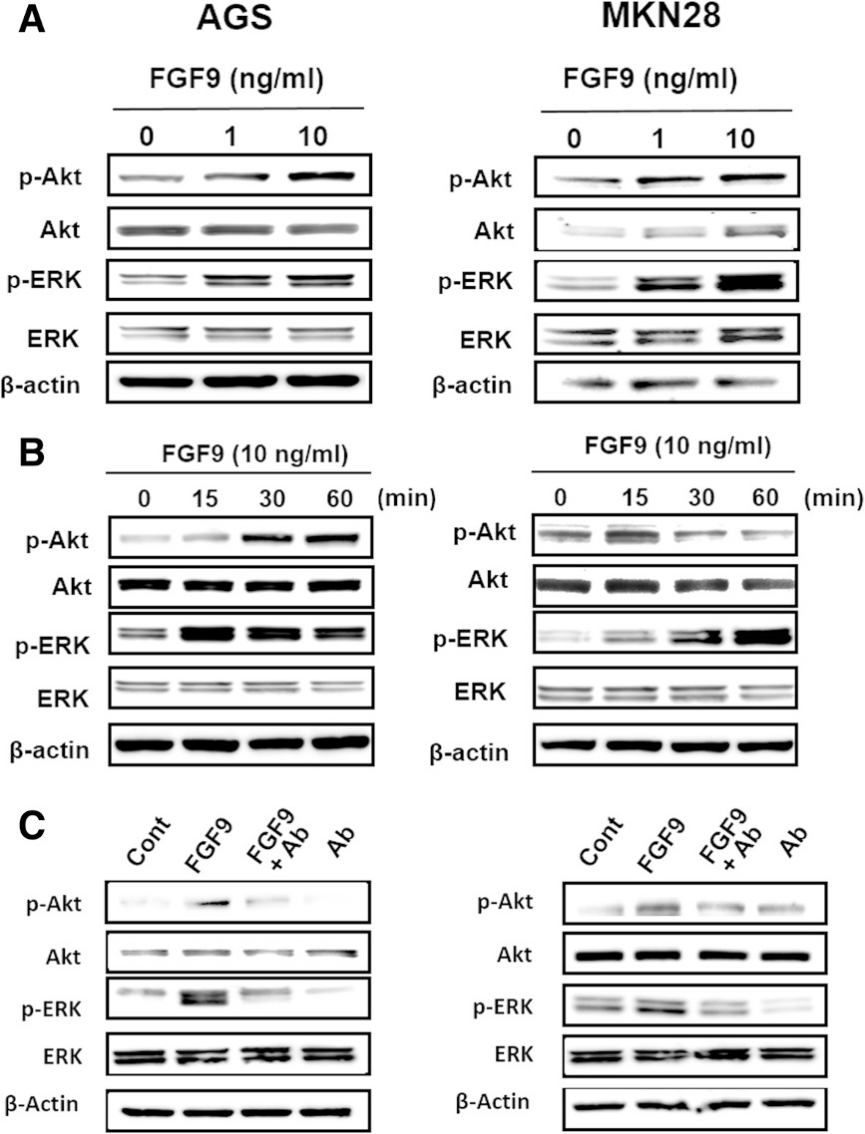
Effect of FGF9 treatment on intracellular signaling in gastric cancer cells. **(A)** Phosphorylation of Akt and ERK in gastric cancer cells treated with FGF9. AGS (4 × 10^5^) and MKN28 (4 × 10^5^) were cultured in six-well plates and treated with various concentrations of FGF9 for 30 min. Extracted protein was analyzed by Western blotting, as described in Materials and Methods. **(B)** Time course change in Akt and ERK phosphorylation in gastric cancer cells treated with FGF9. AGS and MKN28 cells were similarly treated with FGF9 (10 ng/ml) for the indicated times. **(C)** Effect of anti-FGF9 neutralizing antibody on FGF9-induced Akt and ERK phosphorylation in gastric cancer cells. AGS and MKN28 cells were pretreated with anti-FGF9 antibody (Ab; 1 μg/ml) for 45 min and then stimulated with FGF9 (10 ng/ml) for 30 min.

We investigated the effect of FGF9 stimulation on possible pathways including ERK and Akt in gastric cancer cell lines [16]. Expression of both p-Akt and p-ERK was dose-dependently enhanced by FGF9 stimulation in AGS and MKN28 cells (Figure 2A). The enhancement was evident from 15 min after FGF9 (10 ng/mL) treatment in both cell lines (Figure 2B). Moreover, we examined the effect of anti-FGF9 neutralizing antibody on gastric cancer cells and found that the increased expression of p-Akt and p-ERK elicited by FGF9 stimulation was attenuated by concomitant administration of anti-FGF9 neutralizing antibody (Figure 2C).

### Effect of FGF9 on cell proliferation, invasion and anti-apoptosis in gastric cancer cells

**Figure 3:**
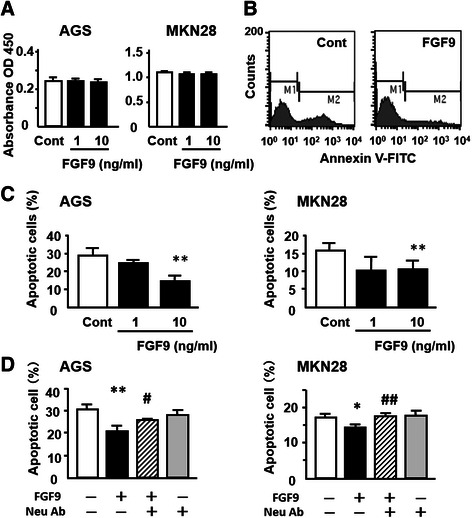
Effect of FGF9 on growth and anti-apoptosis of gastric cancer cells. **(A)** Effect of FGF9 on growth of gastric cancer cells. **(B-D)** Effect of FGF9 on anti-apoptosis capability of gastric cancer cells. **(B)** Representative graphs of FACS analysis using Annexin V-FITC staining. AGS cells were treated with FGF9 (10 ng/ml) and evaluated as described in Materials and Methods. **(C)** Changes in the number of apoptotic AGS and MKN28 cells treated with FGF9. **(D)** Effect of anti-FGF9 neutralizing antibody (Neu Ab; 1 μg/ml) on FGF9 (10 ng/ml)-induced anti-apoptosis in AGS and MKN28 cells. All the results are expressed as the mean ± SD of four samples. Significantly lower than control: *P <0.05, **P <0.01. Significantly greater than the FGF9-treated group: ^#^*P* <0.05, ^##^*P* <0.01.

Since FGF9 is known to have a mitogenic effect on some cell types [17], we first tested the effect of FGF9 on the growth kinetics of gastric cancer cells. However, we found no effect of FGF9 on cell proliferation in the AGS and MKN28 cell lines (Figure 3A).

To further identify the possible role of FGF9 in tumor progression, we examined whether exogenous FGF9 confers an anti-apoptotic effect on gastric cancer cells. FACS analyses revealed that the number of annexin V-positive AGS cells was significantly smaller in the FGF9-treated group than in the control group, and similar findings were obtained in MKN28 cells (Figure 3B and C). Furthermore, this effect of FGF9 was abolished by concomitant administration of anti-FGF9 neutralizing antibody in both cell lines (Figure 3D).

**Figure 4:**
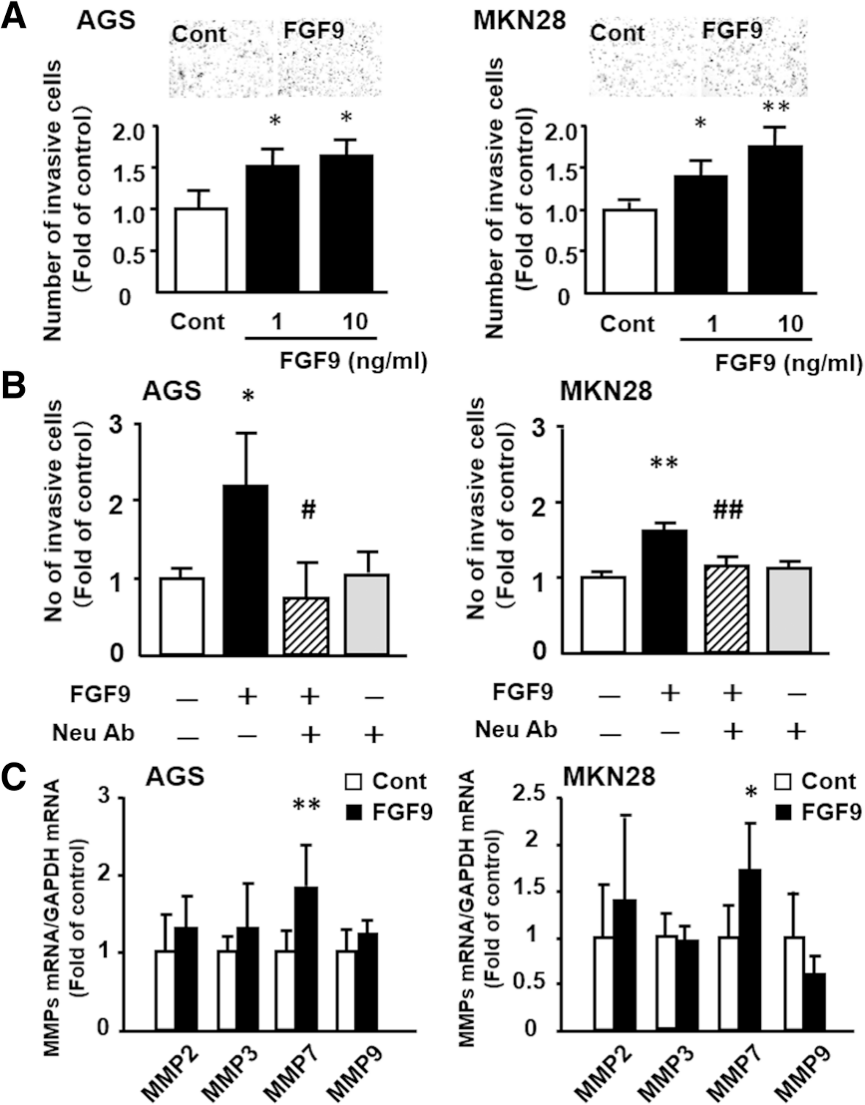
Effect of FGF9 on invasion and MMPs expression of gastric cancer cells. **(A)** Effect of FGF9 on gastric cancer cell invasion. Change in number of invasive AGS and MKN28 cells treated with FGF9 were examined. Photographs showing representative images of invasive gastric cancer cells in the control and FGF9-treated groups. **(B)** Effect of anti-FGF9 neutralizing antibody (Neu Ab; 1 μg/ml) on FGF9 (10 ng/ml)-induced invasion of AGS and MKN28 cells. **(C)** Effect of FGF9 on expression of *MMPs* in gastric cancer cells. All the results are expressed as the mean ± SD of four samples. Significantly greater than control: *P <0.05, **P <0.01. Significantly lower than the FGF9-treated group: ^#^*P* <0.05, ^##^*P* <0.01.

Moreover, we next examined the effect of FGF9 on the invasive ability of gastric cancer cells. When AGS cells were stimulated with FGF9 (1–10 ng/mL), the number of invasive cells was significantly increased (Figure 4A). Similarly, the invasive ability of MKN28 cells was significantly enhanced dose-dependently by FGF9 stimulation (Figures 4A). We then examined whether this pro-invasive effect of FGF9 could be abolished by adding a neutralizing antibody. In both cell lines, after concomitant administration of FGF9 neutralizing antibody (1 μg/ml), the number of invasive cells was significantly decreased in comparison with cells treated with FGF9 alone (10 ng/mL) (Figure 4B). In addition, we examined whether FGF9 induced the expression of MMPs, which play a pivotal role in invasion of various cancers. As shown in Figure 4C, FGF9 stimulation commonly enhanced the expression of *MMP7* in both AGS and MKN28 cells.

### Clinicopathological significance of FGF9 expression in gastric cancer-associated fibroblasts

Of the 20 gastric cancer tissue samples examined, sixteen (80%) was positive for FGF9 expression. Regarding the clinicopathological features, none of the parameters ─ age, gender, tumor location, histological type, or tumor stage ─ had a significant relationship to FGF9 expression (Additional file 2: Table S2).

## Discussion

It is believed that tumor development and progression depend on cross-talk between cancer cells and their surrounding stromal cells. As a major stromal population, “activated” fibroblasts, referred to as CAFs, have been suggested to promote tumorigenesis using various molecular signals, and in the present study we isolated *FGF9* as a novel gene that was overexpressed in CAFs in gastric cancer. Accumulating evidence, including our present data, have shown that CAFs differ from NGFs in terms of not only morphology but also gene expression profiles [18,19], although the underlying mechanisms responsible for these differences are still unclear. On the basis of recent evidence, it is tempting to propose that tumor cells initiate a switch from NGFs to CAFs through some form of signaling [20] or that CAFs originate from cancer cells through epithelial-mesenchymal transition [21,22]. Interestingly, it is widely accepted that the FGF family is crucial for epithelial-mesenchymal transition, not only during development but also in carcinogenesis [9,23]. In the present study, we showed that CAFs are a possible source of FGF9, and that furthermore gastric cancer cells have receptors that are responsive to FGF9, suggesting that FGF9 may be a potential mediator between CAFs and gastric cancer cells.

What is the possible role of FGF9 in the pathogenesis of gastric cancer? Since FGF9 serves as mitogen for prostate or ovarian cancer [17,24], we first investigated the proliferation of gastric cancer cells in the presence of FGF9 *in vitro*. Subsequently, FGF9 failed to promote the proliferation of both AGS and MKN28 gastric cancer cell lines; however, we cannot exclude the possibility that FGF9 may serve as mitogen for other types of gastric cancer cells because FGF9 promotes the proliferation of other gastric cancer cells such as AZ521 or SGC-7901 [25,26]. On the other hand, we clarified in the present study that FGF9 has an anti-apoptotic effect on gastric cancer cells, in accord with the findings of several previous studies [26,27]. In support of this finding, Akt and ERK pathway signaling, crucial for anti-apoptosis, was commonly activated in both gastric cancer cell lines. Furthermore, FGF9-induced Akt and/or ERK phosphorylation and the resulting cell invasion was abolished by blocking FGF9 stimulation, suggesting that FGF9 at least acts as an anti-apoptotic factor.

We also examined whether FGF9 promotes the invasive ability of gastric cancer cells. *In vitro* studies demonstrated that FGF9 treatment increased the number of invading gastric cancer cells, whereas this increased invasive ability was suppressed by adding FGF9 neutralizing antibody. Although it is unclear how FGF9 promotes the invasion of gastric cancer cells, degradation of the extracellular matrix is an important part of this process [28]. In this context, it is accepted that MMPs play a central role in such cell behavior and promote the invasive capacity of various malignancies [29]. Therefore, we screened for MMPs that are linked to FGF9 stimulation and found that MMP7 was potentially involved. Interestingly, recent studies have emphasized the importance of MMP7 in gastric cancer progression, since MMP7 has potential to not only degrade the extracellular matrix but also confer an anti-apoptotic effect on cancer cells [30-32]. Accordingly, in a future study, we intend to focus on the FGF/MMP7 axis in the context of gastric cancer cell invasion.

## Conclusion

In summary, we have shown that gastric CAFs differ from their corresponding NGFs in terms of their gene expression profiles, and propose that FGF9 is a potential molecule that mediates cross-talk between CAFs and gastric cancer cells. Moreover, we have clarified that FGF promotes the anti-apoptosis and invasive capability of gastric cancer cells. Taken together, our data suggest that FGF9 is a possible mediator secreted from cancer-associated fibroblasts that promotes the anti-apoptosis and invasive capability of gastric cancer cells.

## Additional files

Additional file 1: Table S1. Primers for real-time RT-PCR analysis.
Additional file 2: Table S2. Relationship between clinicopathological features and stromal FGF9 expression in patients with gastric cancer.

## Abbreviations

FGF: Fibroblast growth factor
MMP: Matrix metalloproteinase
ERK: Extracellular signal-regulated protein kinase
CAF: Cancer-associated fibroblast
