# Supplementary material for: FGF9 from cancer-associated fibroblasts is a possible mediator of invasion and anti-apoptosis of gastric cancer cells
Source: BMC Cancer. 2015 Apr 30;15:333. doi: 10.1186/s12885-015-1353-3 (PMC4424580; doi:10.1186/s12885-015-1353-3)
Supplement: Additional file 1: Table S1. — Primers for real-time RT-PCR analysis. [file 12885_2015_1353_MOESM1_ESM.doc]

| **Additional file 1: Table S1.** Primers for real-time RT-PCR analysis | | |
| --- | --- | --- |
| MMP2 | Forward | 5’-TGCGGCACCACTGAGGACTAC-3’ |
|  | Reverse | 5’-GGGCTGCCACGAGGAACA-3’ |
| MMP3 | Forward | 5’-ATTCCATGGAGCCAGGCTTTC-3’ |
|  | Reverse | 5’-CATTTGGGTCAAACTCCACTGTG-3’ |
| MMP7 | Forward | 5’-GTGGGAACAGGCTCAGGACTATCTCAA-3’ |
|  | Reverse | 5’-CACATCTGGGCTTCTGCATTATTTCTA-3’ |
| MMP9 | Forward | 5’-AGACCTGGGCAGATTCCAAAC-3’ |
|  | Reverse | 5’-CGGCAAGTCTTCCGAGTAGT-3’ |
| GAPDH | Forward | 5’-GAGTCAACGGATTTGGTCGT-3’ |
|  | Reverse | 5’-TTGATTTTGGAGGGATCTCG-3’ |
|  |  |  |
