# Supplementary material for: FGF9 from cancer-associated fibroblasts is a possible mediator of invasion and anti-apoptosis of gastric cancer cells
Source: BMC Cancer. 2015 Apr 30;15:333. doi: 10.1186/s12885-015-1353-3 (PMC4424580; doi:10.1186/s12885-015-1353-3)
Supplement: Additional file 2: Table S2. — Relationship between clinicopathological features and stromal FGF9 expression in patients with gastric cancer. [file 12885_2015_1353_MOESM2_ESM.doc]

| **Additional file 2: Table S2.** Relationship between clinicopathological features and stromal FGF9 expression in patients with gastric cancer | | | | |
| --- | --- | --- | --- | --- |
|  | Features | FGF9 negative  (n = 4) | FGF9 positive  (n = 16) | *P*-value |
| **Gender** | |  |  | NS |
|  | Man | 1 | 11 |  |
|  | Woman | 3 | 5 |  |
| **Age (yr, mean ± SE)** | | 58.5 ± 9.8 | 65.5 ± 3.0 | NS |
| **Tumor location** | |  |  | NS |
|  | Lower | 2 | 6 |  |
|  | Mid | 2 | 5 |  |
|  | Upper | 0 | 5 |  |
| **Lauren’s classification** | |  |  | NS |
|  | Intestinal type | 0 | 7 |  |
|  | Diffuse type | 4 | 9 |  |
| **Stage** | |  |  | NS |
|  | I | 0 | 1 |  |
|  | II | 1 | 2 |  |
|  | III | 3 | 11 |  |
|  | IV | 0 | 2 |  |
| NS, not significant by Chi-squared analysis. | | | | |
